# Draft Genome Sequence of *Rheinheimera* sp. Strain SA_1 Isolated from Iron Backwash Sludge in Germany

**DOI:** 10.1128/genomeA.00853-16

**Published:** 2016-08-18

**Authors:** Josephin Schröder, Burga Braun, Karsten Liere, Ulrich Szewzyk

**Affiliations:** a Chair of Environmental Microbiology, Technische Universität Berlin, Berlin, Germany; b Services in Molecular Biology GmbH (SMB), Rüdersdorf, Germany

## Abstract

*Rheinheimera* sp. strain SA_1 is an iron-depositing bacterium for which we report a draft genome sequence. Strain SA_1 was isolated from iron backwash sludge of a waterworks in Germany. The Illumina MiSeq technique was used to sequence the genome of the strain.

## GENOME ANNOUNCEMENT

To date, 19 different *Rheinheimera* type strain species are described, which are ubiquitously distributed all over the world and occur in soil (1), freshwater (2), and marine (3) habitats. However, none of the *Rheinheimera* species have been associated with iron or manganese depositions until now. *Rheinheimera* sp. strain SA_1 was isolated from backwash sludge of the Stolpe/Borgsdorf waterworks in Germany after the removal of ochrous depositions from pipes. Reddish-brown colonies developed on modified *Leptothrix* stain medium (4) (deionized water was replaced by sterile well water). These were isolated on medium by a sequential dilution series of suspended iron backwash sludge incubated for seven days at 10°C. Iron and manganese deposition of the strain was confirmed according to Schmidt et al. (5). Strain SA_1 is a rod-shaped Gram-negative bacterium with a length of 2 to 4 µm and a width of 0.8 µm.

The strain was grown on modified LSM2 medium (6), and total genomic DNA was extracted using the GeneMATRIX soil DNA purification kit (Roboklon, Berlin, Germany). The paired-end library was prepared using the TruSeq DNA HT sample prep kit (Illumina Netherlands, Eindhoven, The Netherlands). The genome was sequenced on an Illumina MiSeq sequencer by using the MiSeq reagent kit version 3 (2 × 300 bp), resulting in 4,421,476 reads. For read trimming, base correction and *de novo* assembly were done with SPAdes 3.5 and the Geneious 8 software. The draft genome included 22 contigs. The shortest sequence was 546 bp, and the longest sequence was 1,518,030 bp. The total size of the draft genome was 5,136,701 bp, with a G+C content of 49.3%.

Genome annotation by the NCBI Prokaryotic Genome Annotation Pipeline version 3.2 revealed 4,187 coding DNA sequences (CDS) for proteins, 71 tRNAs, four noncoding RNAs (ncRNAs), and 47 pseudogenes. Further analysis of the annotated three complete 5S, one complete 16S, one complete 23S, and two partial 16S genes gave rise to a genome model with two complete rRNA operons, each with one copy of 5S, long subunit (LSU), and short subunit (SSU). The two 16S rRNA genes are identical to each other, and BLAST searches using the Nucleotide collection (nr/nt) database (7) revealed the *Rheinheimera* sp. F8 genome (8) as the closest relative to strain SA_1, with an identity of 98%. Furthermore, a type I-E clustered regularly interspaced short palindromic repeat (CRISPR) array was identified, including a CRIPSR unit consisting of 46 repeats spanning 45 spacers and seven genes coding for type I-E CRISPR-associated proteins (Cas1, Cas2, CasA-E, and a CDS coding for a DNA binding protein.

### Accession number(s).

This whole-genome shotgun project has been deposited at DDBJ/ENA/GenBank under the accession no. LXWK00000000. The version described in this paper is version LXWK01000000.
